# The *Eucalyptus* genome integrative explorer (EucGenIE): a resource for *Eucalyptus* genomics and transcriptomics

**DOI:** 10.1186/1753-6561-5-S7-O49

**Published:** 2011-09-13

**Authors:** Charles Hefer, Eshchar Mizrachi, Fourie Joubert, Alexander Myburg

**Affiliations:** 1 Bioinformatics and Computational Biology Unit, Department of Biochemistry, University of Pretoria, Pretoria, South Africa; 2 Department of Genetics, Forestry and Agricultural Biotechnology Institute, University of Pretoria, Pretoria, South Africa

## Background

The fast growth and good wood properties of *Eucalyptus* tree species and hybrids make them excellent renewable sources of fiber for pulp and paper production, and woody biomass for bioenergy production. Our research is aimed at understanding the genetic regulation of wood formation in eucalypts, with a focus on transcriptomes, regulatory sequences and gene families involved in secondary cell wall biosynthesis.

## Methods

We have performed deep mRNA sequencing (using Illumina RNA-Seq technology) of several primary and secondary tissues of three *Eucalyptus grandis* trees with the aim to investigate the transcriptional control of cellulose biosynthesis and wood formation. The transcriptome datasets range from nearly mature xylogenic tissues to immature shoot tips, and consists on average 35 million paired-end 80bp short reads. The Illumina short reads were mapped to the latest *Eucalyptus grandis* genome sequence (DOE-JGI v. 1.0, http://www.phytozome.net), and the set of predicted gene models provided by the Joint Genome Institute (JGI). We calculated tissue specific gene expression (FPKM) profiles for each of the ~44 000 predicted genes across the sequenced transcriptome datasets. The results were stored in a relational database for further analysis and analysis of co-expression patterns in the expression profiles. We ultimately aim to identify genes that are differentially expressed and co-regulated during different stages of wood formation.

## Results

We describe the initial development of an Integrative *Eucalyptus* Genome Explorer (EucGenIE), modeled after the poplar resource, PopGenIE (http://www.popgenie.org, Sjödin *et al*, 2009). EucGenIE relies on a relational database system that allows for the efficient storage and retrieval of gene models and expression values from the database, which is then presented to the user in novel and intuitive ways. The web-based front-end makes use of tools available in the Generic Model Organism Database (GMOD, http://gmod.org) toolkit to enrich the query interface as well as result visualization. Links to the Phytozome GBrowse instance provides genomic context to the expressed gene sets. Custom queries allows the user to find genes with similar gene expression profiles across the various datasets, as well as perform bulk searches on sequence features annotated on the gene set. EucGenIE also provides access to common analyses tools, such as homology searching and online clustering tools. The first version of the EucGenIE database and online portal is available at http://eucgenie.bi.up.ac.za with restricted access.
